# Midterm follow-up after embolization of intracranial aneurysms proximal to the circle of Willis with the Silk Vista flow diverter: the I-MAMA registry

**DOI:** 10.1007/s00234-024-03336-9

**Published:** 2024-04-02

**Authors:** Valerio Da Ros, Federico Sabuzi, Francesco D’Argento, Alessandro Pedicelli, Vladimir Gavrilovic, Massimo Sponza, Francesca Di Giuliano, Francesco Biraschi, Marta Iacobucci, Giovanni Grillea, Andrea Bartolo, Mirko Patassini, Paolo Remida, Luca Quilici, Giuseppe Faragò, Marco Varrassi, Nicola Cavasin, Roberto Arpesani, Aldo Victor Giordano, Giuseppe Umana, Francesco Garaci, Roberto Floris

**Affiliations:** 1 Diagnostic Imaging Unit, Department of Biomedicine and Prevention, University of Rome Tor Vergata, Rome, Italy; 2 UOSD Neuroradiologia Interventistica, Dipartimento Di Diagnostica Per Immagini, Radioterapia Oncologica Ed Ematologia, Fondazione Policlinico Universitario A. Gemelli IRCCS, Largo A. Gemelli 8, 00168 Rome, Italy; 3 Division of Vascular and Interventional Radiology, Udine University Hospital, 33100 Udine, Italy; 4 Department of Human Neurosciences, Interventional Neuroradiology, Policlinico Umberto I, “Sapienza” University of Rome, Rome, Italy; 5 UOC Di Neuroradiologia Diagnostica E Terapeutica, Istituto Di Ricovero E Cura a Carattere Scientifico (IRCCS) Neuromed, Pozzilli, IS Italy; 6 Struttura Complessa Di Neuroradiologia, Ospedale San Gerardo, ASST, Monza, Italy; 7 Department of Neuroradiology, AAST Ospedale Papa Giovanni XXIII, Bergamo, Italy; 8 Radiology Department, S. Salvatore Hospital, L’Aquila, Italy; 9 Neuroradiologia, Ospedale Dell’Angelo Mestre, Venice, Italy; 10 Interventional Radiology Unit, Spedali Riuniti Di Livorno, Livorno, Italy; 11 Department of Neurosurgery, Cannizzaro Hospital, Catania, Italy

**Keywords:** Aneurysm, Embolization, Flow diverter, Subarachnoid haemorrhage

## Abstract

**Purpose:**

The aim of this registry was to assess technical success, procedural safety and mid- to long-term follow-up results of the Silk Vista “Mama” (SVM) flow diverter (BALT, Montmorency, France) for the treatment of proximal intracranial aneurysms.

**Methods:**

Between August 2020 and March 2022, data from nine Italian neurovascular centres were collected. Data included patients’ clinical presentation, aneurysms’ size, location and status, technical details, overall complications and mid- to long-term angiographic follow-up.

**Results:**

Forty-eight aneurysms in 48 patients were treated using the SVM. Most aneurysms were small (≤ 10 mm: no. 29, 60%) and unruptured (no. 31, 65%); 13 aneurysms were recurrent after coiling or clipping. 37/48 aneurysms involved the internal carotid artery (77%). Optimal opening and complete wall apposition of the device were achieved in 46 out of 48 cases (96%). Four intra- or periprocedural complications occurred (two thrombotic complications successfully resolved, one cerebellar ischemia, one perirenal hematoma), without new neurological deficit. No significant intra-stent stenosis or stent displacement was observed during follow-up. No FD-related morbidity nor mortality was reported. At midterm (6–12 months) to long-term (> 12 months) follow-up, complete aneurysm occlusion (OKM D) was achieved in 76% of cases. Eighty-eight percent of patients had complete aneurysm occlusion or entry remnant (OKM D + C).

**Conclusions:**

Our experience suggests that the new generation of low-profile SVM flow diverter for the treatment of proximal intracranial aneurysms is safe and effective, with low rates of intraprocedural complications and acceptable mid- to long-term occlusion rate.

## Introduction

Endovascular treatment (EVT) of brain aneurysms with flow diverter stents (FDs) is widespread. Since the introduction of the first FD in 2007 [1], their indications of use quickly expanded, thanks to growing expertise and fast industrial development which provided a large variety of devices in terms of size, design and compatibility.

The availability of low-profile microcatheter and FDs nowadays enables the EVT of intracranial aneurysms at the level and beyond the circle of Willis [2, 3], not amenable for surgical treatment or endovascular embolization with coiling or other techniques; however, the prevalence of unruptured intracranial aneurysms (UIA) proximal to the polygon of Willis is high [4], most involving the internal carotid artery.

The new Silk Vista “Mama” (SVM) FD (BALT, Montmorency, France) is available in the market since August 2020; clinical and angiographic data for the device are still limited in literature, especially regarding mid- to long-term results and outcomes. The aim of this paper is to present the multicentre experience and the available data collected by nine Italian neurovascular centres with the use of the SVM FD for the EVT of proximal intracranial aneurysms.

## Methods

### The silk vista “Mama”

The Silk Vista “Mama” (SVM) is a self-expandable FD designed for the EVT of intracranial aneurysms. It consists of 48 drawn filled tubing (DFT) nitinol/platinum wires with no need for radiopaque markers; the nominal stent diameter ranges from 3.50 to 4.75 mm, and its length ranges between 15 and 40 mm. The device is re-sheathable up to 90% of its length and is delivered through a 0.021″ low-profile microcatheter.

### Study design

The I-MAMA (Italian Silk Vista Mama) registry is a multicentric, observational study involving nine Italian tertiary care Interventional Neuroradiology departments. We performed a retrospective review of all consecutive patients with intracranial aneurysms defined as “proximal” (involving internal carotid artery, vertebral artery or basilar artery) treated with one or more SVMs between August 2020 and March 2022. For all cases, clinical and demographic data were collected, as well as aneurysm status (ruptured or unruptured), size and location, FD size and the use of adjunctive devices.

Primary endpoints of the study were technical success as well as intraprocedural, periprocedural (< 15 days) and postprocedural (> 15 days) safety; technical success included successful opening, apposition or dislocation of the device, whereas safety evaluation involved both neurological and non-neurological complications and adverse events occurred during and after the endovascular treatment, as well as morbidity and mortality rates related to EVT and the stent itself. Morbidity was described as worsening in modified Rankin Scale (mRS) score during follow-up.

Secondary endpoints included efficacy results based on the angiographic occlusion rate of treated aneurysms at mid (6–12 months) to long-term (> 12 months) follow-up. The O’Kelly-Marotta (OKM) [5] scale was used to assess the angiographic occlusion of the aneurysms during follow-up.

No exclusion criteria were considered in terms of aneurysms size, morphology or status. All patients underwent EVT for unruptured aneurysm had a mRS < 2.

### Patients and aneurysms

The study enrolled 48 patients with 48 intracranial aneurysms treated with the SVM flow diverter. Thirty-eight patients were women (79%), in an age range between 25 and 78 years. 37/48 aneurysms involved the internal carotid artery (ICA, from C4 to C7 segments); five in the ICA terminus; six aneurysms were in the posterior circulation, involving the vertebral artery in four cases and the basilar artery in two.

Most aneurysms were small (≤ 10 mm, *n* = 29) and unruptured (*n* = 31); 12 were recurrent after coiling and 1 recurrent after clipping; 4 were ruptured. Among unruptured aneurysms, nine were symptomatic. Aneurysm characteristics are summarized in Table 1.

**Table 1:** Patients and aneurysms

Patients	Results: *n* (%)
Total	48
Age range (years)	25–78
Sex	
Female	38 (79.2)
Male	10 (20.8)
Clinical presentation	
SAH	4 (8.3)
Headache*	6 (12.5)
Cranial nerve palsy*	2 (4.2)
Diplopia	1 (2.1)
Lower limb weakness	1 (2.1)
Asymptomatic	35 (72.9)
Aneurysms’ characteristics	
Total	**48**
Small aneurysms (< 10 mm)	29 (60.4)
Unruptured	31 (64.6)
Recurrent after coiling	12 (25.0)
Recurrent after clipping	1 (2.1)
Acutely ruptured	4 (8.3)
Maximum diameter (mm)	10.1 ± 5.3
Aneurysm type	
Saccular (including recurrent)	42 (87.5)
Dissecting/fusiform	4 (8.3)
Blister	2 (4.2)
Aneurysm location	
ICA C4–C5	4 (8.3)
ICA C6–C7	33 (68.8)
ICA-T	5 (10.4)
V4	4 (8.3)
BA	2 (4.2)

Data expressed as absolute frequencies and percentages

*SAH* subarachnoid haemorrhage, *ICA* internal carotid artery, *BA* basilar artery

^*^One patient experienced both headache and III cranial nerve palsy

### Endovascular procedures

All treatments were performed under general anaesthesia and systemic heparinization. Written informed consent was obtained from all patients.

Dual antiplatelet therapy (DAPT) was administered 5 days before EVT according to institutional protocols for unruptured aneurysms; platelet activity was tested in three patients (VerifyNow PRU Test), and it was not deemed mandatory. Ruptured aneurysms were acutely treated; in these cases, an intravenous antiplatelet drug was administered during the procedure combined with a loading dose of clopidogrel in three out of four cases. Antiplatelet protocols are summarized in Table 2.

**Table 2:** Endovascular treatment and follow-up

Endovascular treatment		
FD removed due to failed opening/detachment inside MC		3 (6.3)
Complete wall apposition		46 (95.8)
Incomplete wall apposition		2 (4.2)
After rescue therapy		0 (0.0)
Adjunctive coiling		10 (20.8)
Platelet activity test		3 (6.3)
Antiplatelet therapy		
ASA + clopidogrel		33 (68.8)
ASA + ticlopidine		11 (22.9)
ASA I.V. bolus + clopidogrel loading dose*		3 (6.2)
Tirofiban (bolus + infusion)*		1 (2.1)
Intra-/periprocedural complications		
Intra-stent clotting		2 (4.2)
Distal embolization		1 (2.1)
Other (perirenal hematoma)		1 (2.1)
Follow-up		
Morbidity	Related to	0 (0.0)
Mortality	EVT	0 (0.0)
DSA follow-up available		33 (68.8)
DSA midterm (6–12 months)		29 (60.4)
DSA long-term (> 12 months)		4 (8.3)
Complete occlusion (OKM D)		25 (75.8)
DSA midterm (6–12 months)		21 (72.4)
DSA long-term (> 12 months)		4 (100.0)
Adequate occlusion (OKM D + C)		29 (87.9)
DSA midterm (6–12 months)		25 (86.2)
DSA long-term (> 12 months)		4 (100.0)
Aneurysms ≤ 10 mm		18 (90%)
Aneurysms > 10 mm		11 (85%)

Data expressed as absolute frequencies and percentages

*FD* flow diverter, *MC* microcatheter, *DSA* digital subtraction angiography

^*^Antiplatelet therapy for ruptured aneurysms

In 10/48 cases (21%), the adjunctive use of coils was required for aneurysms ranging between 11 and 26 mm in largest size; coiling was achieved employing the jailing technique.

Scaffolding with a Leo stent (Balt) was performed in one case of a large unruptured ICA terminus aneurysm (26 mm in largest size).

The size of the SVM was chosen according to parent vessel size and length on the basis of the operator experience; selection of intermediate catheter, microcatheter and microguidewire, as well as other adjunctive devices (i.e. coils, further stents, balloon, etc.), was left to operators’ preferences also.

## Results

Overall, 51 SVM FDs were used and 48 were deployed to treat 48 aneurysms. In one case, the SVM was detached from its pusher inside the microcatheter before reaching the target vessel and was no further used. For ICA aneurysms/ICA terminus (no. 42), deployed stents ranged between 3.5 and 4.75 mm in diameter (average, 4.1 mm ± 0.3 mm) and between 15 and 30 mm in length (average, 20.5 mm ± 4.1 mm); for the EVT of posterior circulation aneurysms (no. 6), stent sizes ranged between 3.5 and 4.75 mm in diameter (average, 4.1 mm ± 0.5 mm) and between 15 and 20 mm in length (average, 20.0 mm ± 4.1 mm).

### Primary endpoints

Once the target vessel is reached, the device opened instantaneously in 46/48 cases (96%); in two cases, the stent was removed due to failed opening or incorrect sizing and was replaced with a second SVM.

**Fig. 1:**
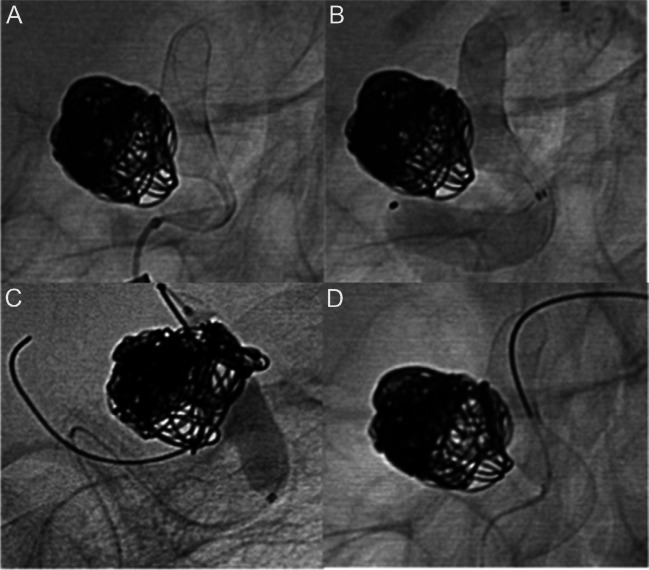
Left para-ophthalmic ICA aneurysm recurrent after coiling. **A**, **B** DSA oblique view during and after deployment of a 4.5 mm × 25 mm Silk Vista, showing incomplete apposition involving the middle segment of the flow diverter. **C** Balloon angioplasty with a 6 mm × 20 mm Eclipse balloon catheter was performed during intravenous infusion of tirofiban, with subsequent full wall apposition (**D**)

Complete wall apposition of the SVM was achieved in 46/48 cases (96%); in two cases, suboptimal opening of the device occurred despite microwire and microcatheter massage, and it was successfully managed intraoperatively in both cases with balloon angioplasty and the adjunctive use of a loading dose of tirofiban (Aggrastat®) in one case (Fig. 1). Incomplete apposition involved the middle and the proximal segment of the SVM, respectively.

**Fig. 2:**
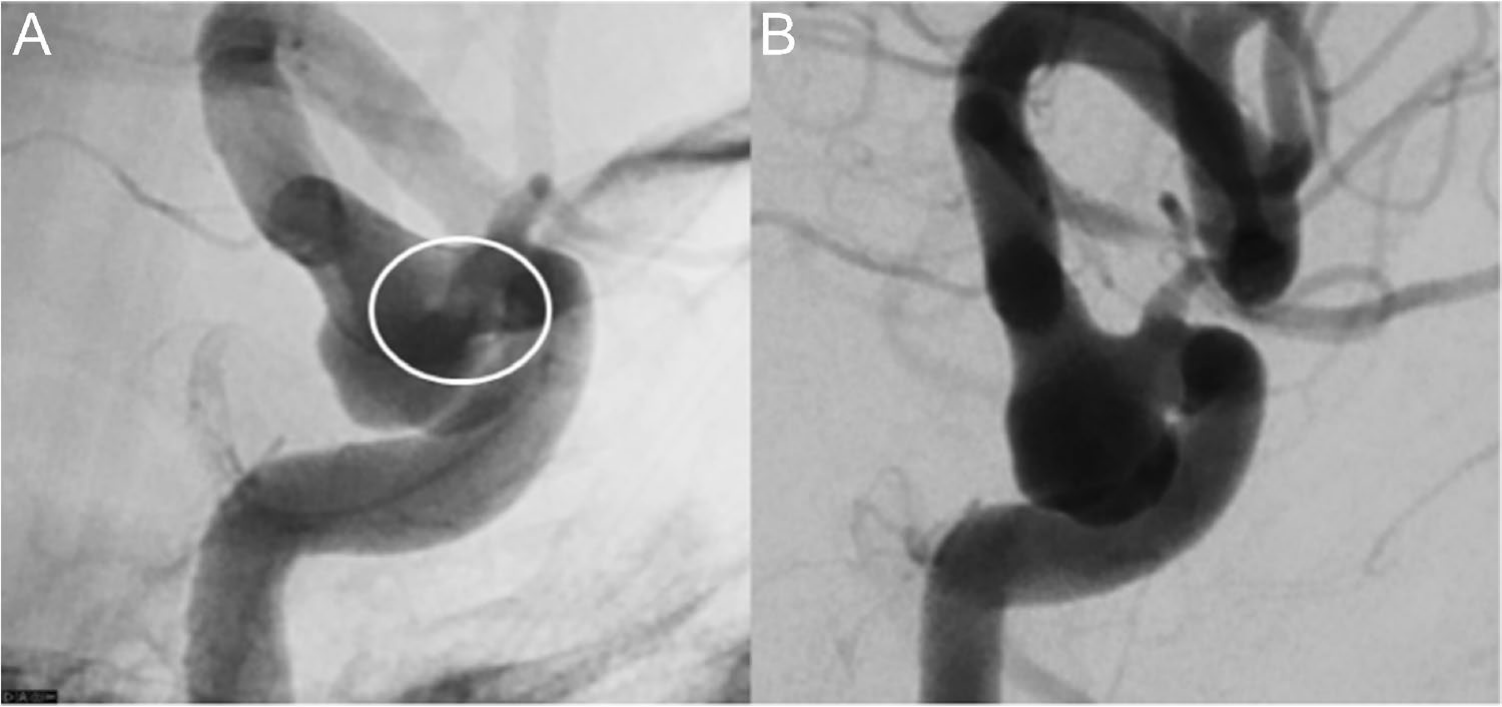
Right para-ophthalmic saccular ICA aneurysm. **A** A small clot developed at the aneurysm neck after deployment of a 4.5 mm × 25 mm Silk Vista. **B** Complete reperfusion after intravenous bolus of tirofiban

Intraprocedural and periprocedural complications (up to 15 days after EVT) were reported in four cases (8%); three were classified as adverse neurological events, resolved with no clinical consequences. These included two cases of intra-stent clotting during EVT of saccular ICA aneurysms (Fig. 2), managed with intravenous administration of tirofiban (Aggrastat®) or abciximab (Reopro®), and cerebellar bilateral ischemic lesions reported in a brain MRI performed 3 days after the EVT of a dissecting aneurysm of the left V4 segment. A perirenal hematoma also occurred during successful embolization of a large ICA cavernous aneurysm; abdomen CT angiography showed no active bleeding, and the hematoma was managed conservatively.

No postprocedural events were reported.

No intraprocedural events were reported when adjunctive techniques (FD-assisted coiling or stent scaffolding) were employed.

Overall morbidity and mortality rates were 2.1% and 2.1%, respectively; one patient had a mRS of 3 at 14-month follow-up due to clinical complications related to the SAH, whereas another patient died during follow-up due to metastatic cancer. EVT- and stent-related morbidity and mortality rates were 0.0% and 0.0%, respectively.

### Secondary endpoints

Both clinical and angiographic midterm (6–12 months) to long-term follow-up (> 12 months) were available in 33/48 patients (69%) and were performed according to institutional protocols; most patients had a midterm follow-up (*n* = 29). Mean follow-up time was 8.1 months (range 6–14 months).

**Fig. 3:**
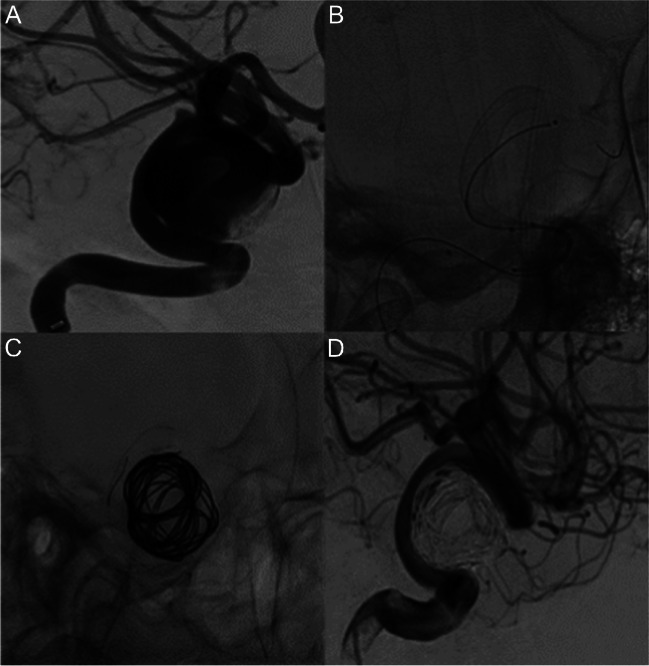
Right unruptured cavernous ICA aneurysm (22 mm, **A**); patient symptomatic for diplopia. DSA oblique views: jailing of the microcatheter inside the aneurysm during deployment of a 4.5 mm × 30 mm Silk Vista (**B**) and subsequent coiling (**C**). Nine-month follow-up DSA (**D**) shows complete aneurysm occlusion (OKM D)

Aneurysm occlusion rates at the latest angiographic follow-up were as follows: OKM D in 25 cases (25/33, 76%) (Fig. 3), OKM C in 4 cases (4/33, 12%), OKM B in 3 cases (3/33, 9%) and OKM A in 1 case (1/33, 3%). Overall, aneurysm complete occlusion or entry remnant (OKM D or C) was reported in 29/33 patients (88%). Angiographic images were self-evaluated by the operators during follow-up.

**Fig. 4:**
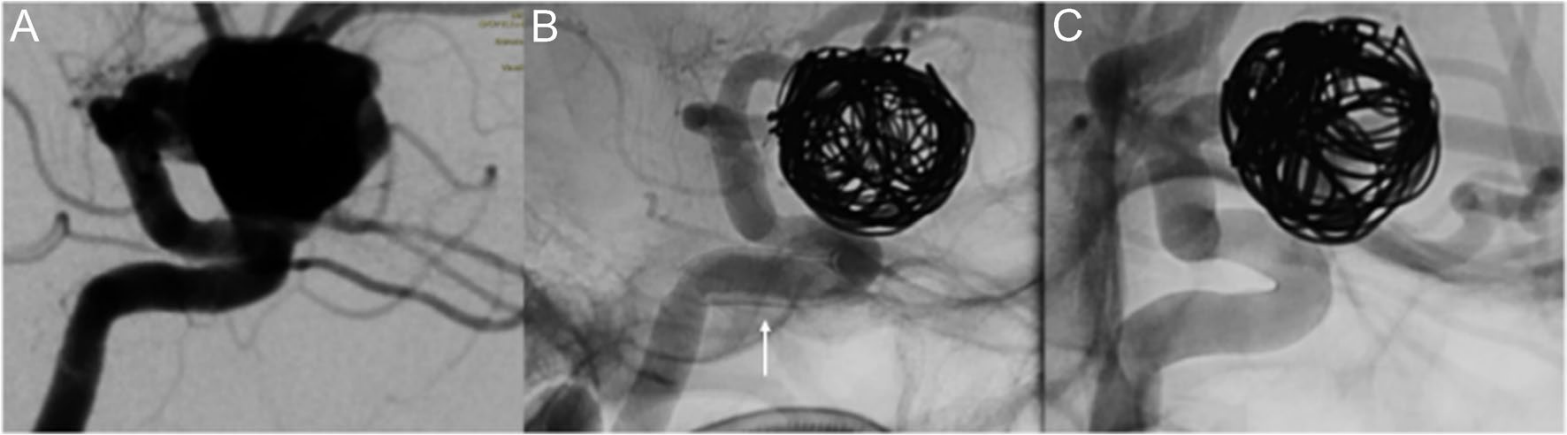
Large left para-ophthalmic ICA aneurysm (14 mm) treated with coils and a Silk Vista 4 mm × 25 mm flow diverter (**A**). At 3-month follow-up (**B**), mild intimal hyperplasia was noted (arrow); complete resolution of intimal hyperplasia after 6 months of dual antiplatelet therapy

Two cases of mild neointimal hyperplasia were detected during angiographic follow-up, with no clinical consequences, and underwent complete resolution after further 6 months of DAPT (Fig. 4); no significant intra-stent stenosis was observed. There were no cases of stent displacement or shortening during follow-up.

No DSA or MRI data were available during follow-up in nine cases (two patients were lost to follow-up due to intervening neoplasm). In 4/6 cases (8%) in which MRI was obtained during follow-up (mean follow-up: 5 months, DSA follow-up not available), complete or near complete occlusion of the aneurysm was achieved.

## Discussion

This is the largest case series of proximal intracranial aneurysms treated with the new generation of low-profile SVM stent, involving nine Italian tertiary Interventional Neuroradiology centres, providing a mid- to long-term clinical and imaging follow-up assessed by DSA.

The SVM is a FD designed for the EVT of intracranial aneurysms involving vessels up to 5 mm in diameter, thus proximal to the circle of Willis, compatible with 0.021″ microcatheters. Many studies and large prospective trials have already evaluated safety and efficacy of FDs [6, 7]; however, development of new technological advances on FD design, composition and coating are constantly in progress. The achievement of a lower profile microcatheter to deliver FDs also in larger vessels seems to be one of the latest technical improvements in the field. In our experience, SVM low profile and design allowed an easier navigability, even in tortuous anatomy demonstrated by the safety in reaching straightforward the target vessel in all the treated patients. Compared with the previous generation of Silk FDs, the SVM has also higher radial force [8], and we observed a complete wall apposition immediately after stent deployment in 46/48 procedures (96%). This result is in line and even slightly superior to previous early experiences with the new generation of Silk Vista reported by Martinez-Galdámez [8] and Pumar [9] (92%, respectively). Moreover, the visibility of SVM seems enhanced.

Despite no restriction is made by the company regarding the 0.021″ microcatheter to be used with SVM, in one case, we observed an early stent detachment from its pusher once inside the microcatheter TREVO 18 (Stryker) before reaching the target vessel; in this case, the SVM was no further used. A possible responsibility of this was attributed to the characteristic of the microcatheters’ hub, since no other episodes occurred using other different microcatheters currently available in the market.

Beyond the technical innovation, due to the large number of centres and operators involved, further considerations were made from this registry. First, as already mentioned, the operators involved found a further improvement of the devices in terms of navigability, and the main reason was attributed to the 0.021″ microcatheters used. Indeed, the feeling of navigation of this type of microcatheters is also commonly employed with stent retrievers during mechanical thrombectomy with which was referred much more. Moreover, all the involved operators agreed that the “unsheathing technique” modality of SVM deployment was easier compared with the previous generation of FD. At the same time, the result obtained in our cohort favourably compared with those of Lubicz et al. [10] with the use of previous generations of Silk FDs in terms of both wall appositions (96% vs 93%) and adequate occlusion during follow-up (88% vs 89%).

Other than the high technical success rate with SVM, we also observed low rates of intraprocedural or periprocedural complications and none determining major adverse events. Three cases of thromboembolic events (6%) were reported in our series, two of which related to intra-stent clotting during the EVT, similarly to those reported by Martinez-Galdámez [8] et al. and consistent with a recent meta-analysis by Florez et al. on the predecessor SILK [11]. Moreover, in our series, both thromboembolic events occurred despite optimal wall apposition of the stent; potential endothelial damage of the stent may have a causative role, as suggested by some authors [12]. As most patients’ platelet activity was not tested (including those who experienced thromboembolic complications), these events might also be due to unsatisfactory response to the DAPT protocols adopted; however, due to the rarity of thromboembolic events occurred in this series, it remains difficult to identify the underlying mechanism.

Overall, in this registry, only one patient experienced asymptomatic cerebellar ischemic lesions during early MRI follow-up. Considering the 0.0% of thromboembolic events in the Silk Vista cohort described by Pumar et al [9], the use of low-profile FDs could still be one of the possible explications of this very low complication rates, but these results should be confirmed by larger series.

The SVM was also successfully employed in four cases of ruptured intracranial aneurysms in our series, two of which were blister-like aneurysms of the anterior and posterior circulation, respectively, and no intraprocedural complications were reported. In the acute setting, complete ruptured aneurysm occlusion occurred in ¾ of cases at mid- to long-term follow-up (75%, OKM D), and all patients improved their mRS score, although one patient showed an mRS score of 3 not related to the stent itself.

Thanks to the extended follow-up, differently from the previous reports about the new generation of Silk Vista, we aimed at evaluating not only postprocedural events in a short-term period but also the occurrence of complications during longer angiographic follow-up, including the frightening “fish mouth” effect mostly described with the new generation of FD. Regarding this, no significant adverse events were reported and no stent modifications were observed, except for the detection of mild neointimal hyperplasia in two cases. The latter ran asymptomatic and resolved without clinical consequences with prolonged DAPT for further 6 months. Despite no signs of neointimal hyperplasia were reported by Pumar [9] with the use of the new generation of the Silk Vista, Caroff et al. [13] already described this phenomenon more frequently associated with the use of first-generation Silk stent rather than other FD at short-term follow-up. Due to the few numbers observed and, even more important, to the use of different DAPT protocols used in this multicentre experience, further studies are needed to better assess its prevalence in short- to long-term follow-up with the new generation of SVM.

The main limitations of this study include its retrospective design, the self-evaluation of clinical and angiographic follow-up and the heterogeneity of clinical practice among different centres involved, especially in terms of used DAPT and technical features of the EVT; moreover, at the time of the study, a DSA follow-up was available in 33/48 patients. Despite this, the I-MAMA registry still represents the largest series with mid- to-long term follow-up with the use of SVM FDs for the EVT of proximal unruptured and ruptured intracranial aneurysms.

## Conclusions

In conclusion, the SVM is a safe and effective device for the endovascular treatment of complex proximal intracranial aneurysms. Its low-profile and technological advancement seems to further improve the device navigability in difficult anatomies, with low adverse events and high rates of complete aneurysm occlusion at mid- to long-term angiographic follow-up.
